# *Homo sapiens* could have hunted with bow and arrow from the onset of the early Upper Palaeolithic in Eurasia

**DOI:** 10.1016/j.isci.2025.114270

**Published:** 2025-12-18

**Authors:** Keiko Kitagawa, José-Miguel Tejero, Reuven Yeshurun, Rudolf Walter, Hannah Huber, Robin Andrews, Nico Magliozzi, Luc Doyon

**Affiliations:** 1 Senckenberg Centre for Human Evolution and Palaeoenvironment, University of Tübingen, Tubingen, Germany; 2 Institute of Archaeological Sciences, Department of Geosciences, University of Tübingen, Tübingen, Germany; 3 SERP (Seminari d’Estudis I Recerques Prehistòriques) Barcelona University, Montalegre 6, Barcelona 08001, Spain; 4 Department of Evolutionary Anthropology, University of Vienna, Vienna, Austria; 5 Human Evolution and Archaeological Sciences (HEAS) of the University of Vienna, Vienna, Austria; 6 Zinman Institute of Archaeology and School of Archaeology and Maritime Cultures, University of Haifa, Mt. Carmel, Haifa 3103301, Israel; 7 Working Group of Prehistory and Quaternary Ecology, Department of Geosciences, University of Tübingen, Tübingen, Germany; 8 Université de Bordeaux, MCC, CNRS UMR5199 PACEA, Bât. B2, Allée Geoffroy Saint Hilaire, CS50023, Pessac CEDEX 33615, France

**Keywords:** anthropology, archeology, evolutionary biology, experimental archaeology

## Abstract

The evolution of projectile technology remains a central topic in palaeoanthropological discussions on prey acquisition, subsistence strategies, and interpersonal violence. A linear technological development is traditionally assumed from handheld spears, spear-thrower and spears (darts), to bow-and-arrows throughout the Palaeolithic, although recent studies argue for a more complex scenario. Here, we combine experimental ballistic with use-wear and morphometric analyses to investigate whether Aurignacian (c. 40–35 kya) osseous projectile points represent a diverse hunting strategy, i.e., whether some armatures were hafted on arrows rather than on spears. Our results suggest that breakage patterns depend more on the raw material and size of the armature than its specific launching mechanism. Variation in damage types and sizes recorded for arrowheads falls within that observed for spears. Thus, we suggest that Aurignacian hunting gears represent diverse weaponry technologies that possibly include both spear-thrower-and-spear and bow-and-arrows from the onset of the early Upper Palaeolithic.

## Highlights

• We tested whether osseous projectile points were arrowheads using experimental ballistics
• Variation in damage type and size of arrowheads falls within that observed for spears (darts)
• Humans may have used bow-and-arrow in the Early Upper Palaeolithic as well as spear-throwers

## Graphical abstract

**Figure:**
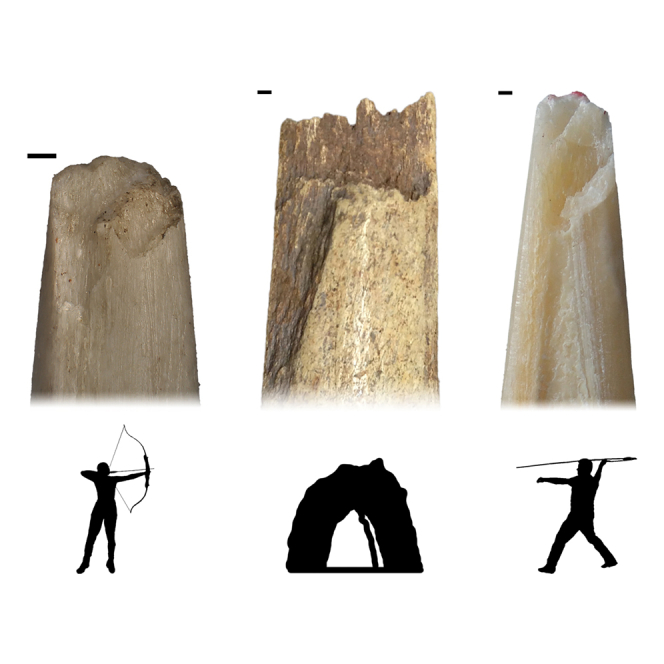

## Teaser

Anthropology; Archeology; Evolutionary Processes; Evolutionary Biology; Experimental Archaeology

## Introduction

The Palaeolithic origin and development of projectile technology, which refers to composite tools with a launching device and a weapon, remains a central topic in palaeoanthropological and archaeological discussions on prey acquisition, subsistence strategies, and the emergence of warfare. Traditionally, the general evolutionary trajectory for mechanically propelled weapons assumed a linear technological evolution from handheld spear, spear-thrower and spear (or dart) to bow-and-arrow throughout the Palaeolithic. While part of this trajectory seems to reflect changes in the complexity of tools from simple to composite weapons, this hypothesis is supported by a limited archaeological record, which is heavily biased by the poor preservation of organic remains such as the wooden components used in the manufacture of Palaeolithic weapons.

In recent decades, several studies aimed to establish whether projectile types could be inferred from the morphometry of lithic armatures or the impact damages they bear. These investigations gave rise to a more complex, non-linear view on the development of hunting technologies throughout the Palaeolithic.^1^

To this day, however, the role of osseous projectile points in this development remains unclear. Considering the recent revisions to the traditional model of weapon development, the present study seeks to test this assumption by comparing the efficiency of different delivery mechanisms, material, and size of projectile weapons. It also aims to establish whether spears (otherwise known as darts), which are delivered by spear-throwers, and arrowheads delivered by bows can be distinguished based on the morphometry of an osseous armature and the impact damages present on it.

### Background

Direct evidence for hunting weapons is rare in the archaeological record. Prehistoric hunting weapons range from handheld thrusting spears, which are effective for close-range hunting, to spear-on-spear-thrower as well as arrow-on-bow, which are used for medium or long-range hunting. The earliest appearances of such tools are wooden spears and throwing sticks that date to 337–300 thousand years before present (ka) in Europe.^2^^,^^3^^,^^4^ Antler objects interpreted as spear-thrower hooks begin to be documented in Upper Solutrean contexts (c. 24.5–21 ka), e.g., Combe Saunière 1, Dordogne, and Grotte du Placard, Charente,^5^^,^^6^ and they become more visible in the Magdalenian (from c. 21 ka) from Southwestern France with almost a hundred specimens.^7^^,^^8^ Meanwhile, bow-and-arrow technology is only found in exceptionally well-preserved contexts at the Final Palaeolithic sites of Mannheim-Vogelstang and Stellmoor, Germany,^9^^,^^10^^,^^11^ dated to circa 12 ka, and at the early Mesolithic site of Lilla Loshults Mosse, Sweden (c. 8.5 ka)^12^ making it much younger than other projectile technologies. Together, these discoveries support the traditional linear view on the development of hunting strategies, from hand-held simple weapons used in close-range hunting to more complex launching mechanisms allowing the acquisition of prey at a longer distance.^6^^,^^13^^,^^14^^,^^15^^,^^16^^,^^17^^,^^18^^,^^19^ Projectile impact marks (PIM) on archaeological animal and human bones, while sporadic, suggest a similar scenario in which a few PIM resulting from thrusting or throwing a short-range spear appear in the Middle Palaeolithic and Middle Stone Age, alongside cases of PIM from simple wooden spears.^9^^,^^20^^,^^21^^,^^22^ In the late Upper Palaeolithic and the Mesolithic, PIM that can be attributed to small and composite projectiles, such as bow and arrow, are better manifested.^23^^,^^24^^,^^25^

The vast majority of objects interpreted by archaeologists as weapon components are lithic or osseous projectile points recovered at sites in different geographic and cultural contexts throughout the Palaeolithic. These implements show high diachronic variability (e.g.,^26^^,^^27^^,^^28^^,^^29^) and a certain degree of standardization which made them ideal candidates to establish the chronological framework for the succession of pre- and proto-historic cultural entities in various regions of the globe (e.g.,^30^^,^^31^^,^^32^^,^^33^^,^^34^). Over the last few decades, substantial experimental and ethnographic research was undertaken to establish criteria to infer the weapon type from the morphometric variability of projectile armatures^14^^,^^35^^,^^36^^,^^37^^,^^38^ and from the impact damage they bear (e.g.,^39^^,^^40^^,^^41^^,^^42^^,^^43^^,^^44^^,^^45^^,^^46^). Potential use of spear-throwers and darts is now suggested in Eurasia from the Late Glacial Maximum (LGM) cold event (c. 29–19 ka) (e.g.,^19^^,^^47^^,^^48^^,^^49^^,^^50^^,^^51^^,^^52^). Some authors even argue for an early emergence of bow-and-arrow technology among *Homo sapiens*, specifically during the African Middle Stone Age, which has been demonstrated by experimental study on fracture, use-trace and residue analyses,^15^^,^^17^^,^^41^^,^^44^^,^^53^^,^^54^^,^^55^ and from around 50–45 ka in several regions and diverse chrono-cultural contexts from Western Europe to the Near East and Eastern Asia (e.g.,^56^^,^^57^^,^^58^^,^^59^^,^^60^^,^^61^). One recent study argues for even earlier dates (80 ka) in Russia.^62^ These reports, therefore, question the traditional evolutionary scenario for the emergence of projectile technology and argue for a more complex and nonlinear development.

Most experimental sessions designed to distinguish prehistoric weapon types, i.e., arrows versus spears, entailed using replicas of lithic armatures rather than osseous ones. Likewise, data on large ethnographic collections rely on the dimension of knapped stone or metal armatures that are hardly comparable to osseous ones. Indeed, bone projectile points lack the lacerating properties of their counterparts made of stone or metal that increases the potential of inflicting a lethal wound to the prey^63^^,^^64^ unless poison is applied on osseous armatures.^38^ Osseous projectile points developed contemporaneously in various regions of the Old World in widely diverse environmental conditions between 40 and 35 ka^59^^,^^65^^,^^66^^,^^67^^,^^68^^,^^69^^,^^70^^,^^71^^,^^72^^,^^73^^,^^74^^,^^75^^,^^76^^,^^77^^,^^78^^,^^79^^,^^80^^,^^81^^,^^82^ and it is generally assumed they were hafted on spears and darts. Some exceptions include the specimens found at Fa-Hien Lena, Sri Lanka^59^ or in MSA contexts at Sibudu Cave or Klasies River, South Africa^54^^,^^55^ that were typologically attributed to arrowheads based on their small size or on incipient fractures observed by computed tomography scan present in their osseous matrix and interpreted as resulting from impact.

In the present study, we report a systematic experiment addressing the question of weaponry systems based on osseous projectiles. We focus on the Upper Palaeolithic osseous projectile implements, including the split- and massive-based points made of antler and bone, which were mostly found in Aurignacian contexts in Europe and the Levant between c. 40–33 ka (but see^56^^,^^83^). Our aim is to investigate whether it is possible to establish the type of weapons on which Aurignacian osseous projectile points were assembled from the use-wear patterns they bear and their morphometry.

## Results

### Overview

We performed a total of 191 shots (delivered 78 times using crossbow, 34 bow, 79 spear-thrower) with the 60 projectiles (19 arrows and 41 spears) manufactured for the shooting experiments (Data S1). In terms of accuracy, 32 shots out of the 41 (78%) made with arrows hit the target. For spears, 82 shots out of 150 (55%) hit the target. Arrows were shot between one and four times before being discarded while spears were shot between 1 and 14 times. An average of 2.1 shots was required to induce damage on the arrows compared to 2.6 shots for spears. The proportion of shots resulting in hafting damage amounted to 18.3%, i.e., 26.8% for arrows and 16% for spears. In four instances, i.e., one spear and three arrows, the armature got stuck in a long bone, scapula, or vertebrae.

### Efficiency

**Figure 1:**
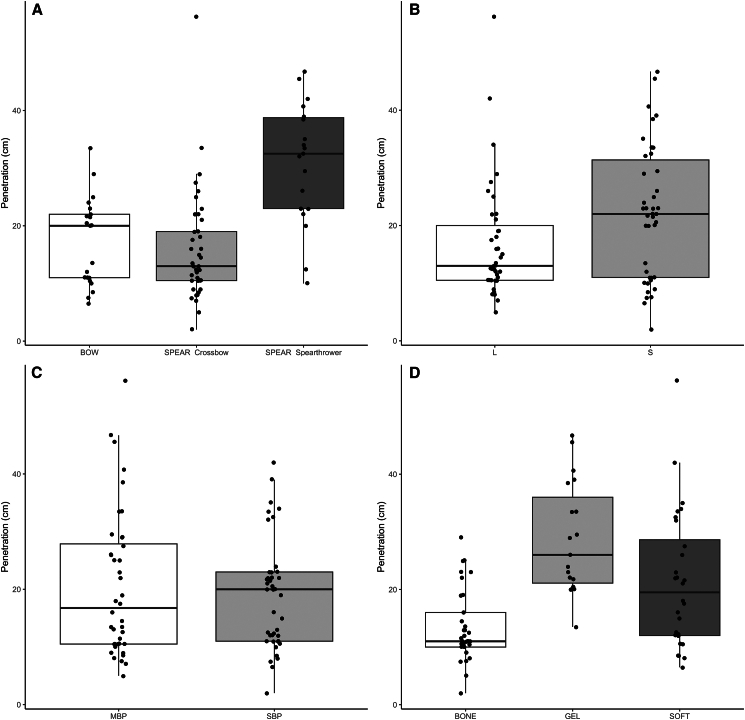
Distribution of the penetration values recorded during the shooting experiment (A) Delivery method. (B) Armature’s size (L = large; S = small). (C) Point type (MBP = massive-based points; SBP = split-based points). (D) Impacted tissue. See Data S1 for details. The box and the line in the plot represent the overall range of the data.

To establish whether arrows and spears would be efficient projectiles that could induce a lethal wound to the prey, the depth of penetration recorded during the shooting experiment was compared with the type of tissue impacted, the armature type and size, the delivery method and its kinetic energy. For the 82 shots that penetrated the target, an average depth of 19.6 cm was recorded. The depth of penetration for arrows ranged between 6.5 and 33.5 cm with an average of 16.4 cm, which is significantly lower than that of spears delivered with a spear-thrower (Student’s *t = −*4.6499, df *=* 32.757, *p* value *= <*0.0000) which ranged between 10 and 46.7 cm with an average of 30.8 cm (Figure 1A). Interestingly, spears delivered with a crossbow reached depths of penetration that fall within the range documented for arrows. The bimodal distribution recorded for arrows is explained by the experimental setting. The lower mode corresponds to specimens impacting bones that were encased in the ballistic gel while the higher one reflects the depth values for arrows piercing through the gel blocs. This pattern contrasts with the continuous distribution recorded for spears that were mostly shot onto suspended carcasses. Although the number of arrow shots on carcasses is insufficient to reach definite conclusions, the available data suggests that the depth values would likely be distributed continuously rather than bimodally if more shots were performed on carcasses, therefore behaving similarly to spears. Projectiles armed with a smaller armature tend to penetrate significantly deeper in the target than those armed with larger ones (Student’s *t =* −2.2003, df *=* 78.842, *p* value = 0.03071; Figure 1B). There is, however, no significant differences for the depth of penetration when the type of armature (massive-based points vs. split-based points) is considered (Student’s *t =* 0.68939, df *=* 72.528, *p* value = 0.4928; Figure 1C). As expected, the depth of penetration varies significantly depending on the impacted tissue (ANOVA: *F*_(2,77)_ = 16.51, *p* value = <0.00001; Figure 1D). Depth values are significantly higher for soft animal tissues compared to hard ones (Tukey’s HSD: adjusted *p* value = 0.00502, 95% CI = [2.04,13.56]). An additional significant difference in depth of penetration was found between soft animal tissues and ballistic gel (Tukey’s HSD: adjusted *p* value = 0.02394, 95% CI = [−14.15,−0.82]).

**Figure 2:**
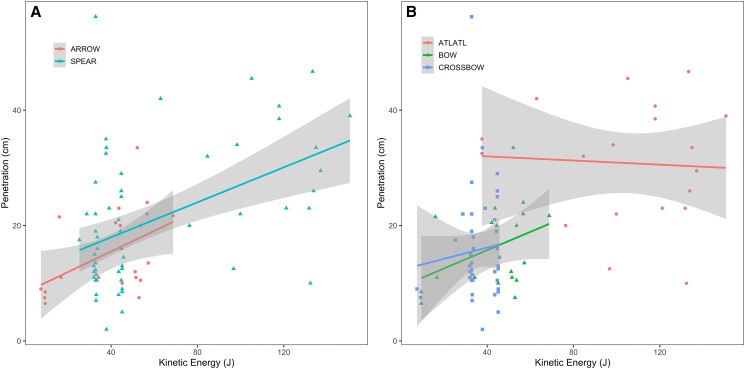
Relation between the depth of penetration as a function of kinetic energy (J) (A) Projectile type. (B) Delivery method. Linear trends and 95% confidence intervals by category. See Data S1 for details.

Comparison between the penetration depth and the kinetic energy suggests a positive correlation which is statistically significant between these two variables (*R*^2^ *=* 0.51, Pearson’s *t =* 5.142, df *=* 77, *p* value *=* 2.01e-06). When the delivery method is considered, the range of values recorded for arrows overlaps with the lower range of variation recorded for spears (Figure 2A). However, most of the lower values recorded for the spear correspond to shots delivered with the calibrated crossbow (Figure 2B). Interestingly, the average kinetic energy of a projectile shot by an experienced individual (Joachim Martz) reached 50.9 J for arrows and 124.3 J for spears, while the kinetic energy for spears launched with the crossbow reached on average 37.5 J. Thus, the efficiency of spear- and arrow-delivered projectiles may be similar; however, its variability is conditioned on many variables that cannot be controlled in an archaeological context.

### Shooting damages on armatures

**Figure 3:**
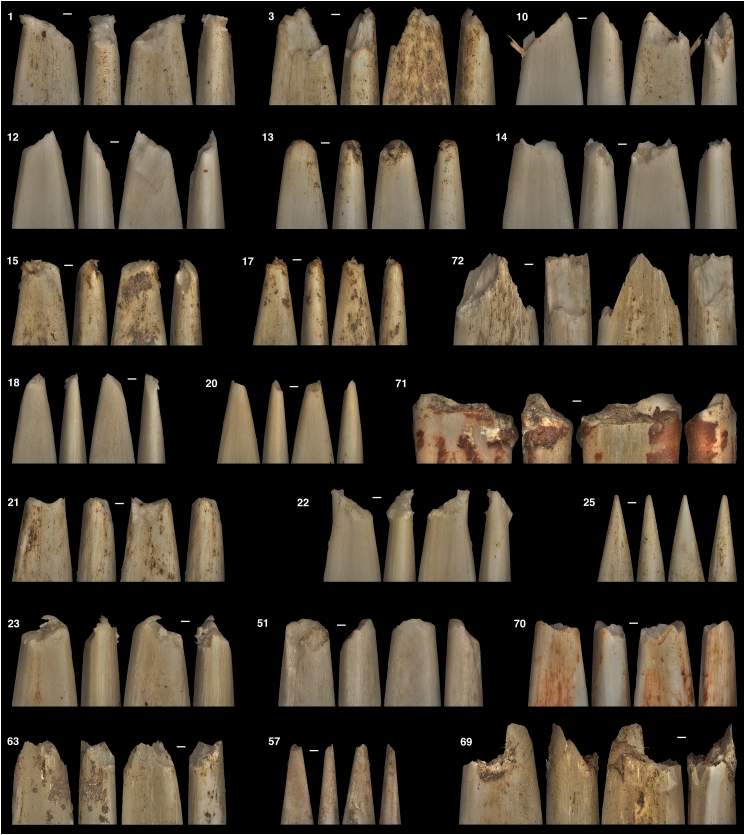
Sample of distal damages produced when shooting Aurignacian osseous projectile points hafted Numbers refer to the specimen ID in the Data S1. Scales = 1 mm.

A total of 87 instances of damage were recorded during the shooting experiments, among which 19 were observed on armatures hafted on arrows and 68 on armatures hafted on spears, including 30 on those delivered with a crossbow (Figure 3). Roughly one-third of the time (34.5%), the damage entailed a compression, deformation or split of the armature’s apex but did not result in the removal of osseous material from the tip. Often, shooting damages proved to be complex, combining multiple categories of detachment of osseous material and compression of what remained from the armature’s apex especially after the projectiles were shot multiple times (Table 1). To simplify the comparison, distal damages that caused the detachment of osseous material fell in three broad categories: tongue fracture (19.5%) includes both simple and double tongue fractures with or without compression; hinge fracture (14.9%) encompasses damages presenting at least one tongue with a hinge termination but no tongue with step termination; step fracture (29.9%) refers to damages presenting at least one tongue with a step termination. A sawtooth fracture was observed in a single instance (1.2%).

**Table 1:** Absolute and relative frequency of damage types observed on arrow and spear armatures

Damage type	Arrow	Arrow %	Spear (spear-thrower)	Spear % (spear-thrower)	Spear (crossbow)	Spear % (crossbow)	Total
Compression	4	21.05%	9	23.68%	7	20.00%	19
Deformation	1	5.26%	5	13.16%	3	10.00%	9
Split	1	5.26%	1	2.63%	–	–	2
Tongue	1	5.26%	2	5.26%	2	6.67%	5
Tongue + compression	5	26.32%	4	10.53%	1	3.33%	10
Double tongue	–	–	–	–	1	3.33%	1
Double tongue + compression	1	5.26%	–	–	–	–	1
Tongue + hinge	–	–	1	2.63%	–	–	1
Hinge	2	10.53%	–	–	2	6.67%	4
Hinge + compression	1	5.26%	7	18.42%	–	–	8
Step	1	5.26%	4	10.53%	8	26.67%	13
Step + compression	2	10.53%	1	2.63%	3	10.00%	6
Double step + compression	–	–	1	2.63%	–	–	1
Step + hinge	–	–	1	2.63%	3	10.00%	4
Step + hinge + compression	–	–	1	2.63%	1	3.33%	2
Sawtooth	–	–	1	2.63%	–	–	1
Total	19	–	38	–	30	–	87

See Data S1 for details.

**Figure 4:**
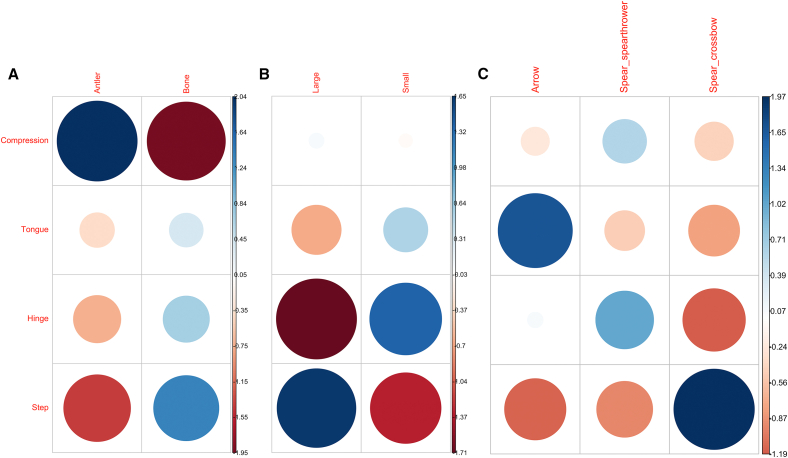
Pairwise comparisons between the distal damage categories (A) The raw material of the armature. (B) Its size. (C) The projectile type. The size of the circle is proportional to each pair’s residual contribution to the χ^2^ value (or Fisher’s exact test in the case of C) while colors refer to their standard error. See Tables 1, 2, and 3 and Data S1 for details.

The relative frequency of the distal damage categories was compared with the raw material and size of the armature as well as the type of projectile used. A substantial overlap was documented in the proportion of damages recorded in all cases. However, when raw material is considered, compression is more frequently observed on antler armatures than bone ones, while the latter are more prone to, in decreasing order, step, hinge and tongue fractures (χ^2^ = 13.075, df = 3, *p* value = 0.004477; Table 2; Figure 4A). When size is concerned, large armatures appear more prone to step fractures while small specimens are more likely to display hinge and tongue fractures (χ^2^ = 10.019, df = 3, *p* value = 0.01841; Table 3; Figure 4B). Finally, although there is a substantial overlap on the frequency of damage recorded for arrows and spears delivered with spear-thrower, the former displays a higher proportion of tongue fractures and a lower proportion of step fractures while compression damages and hinge fractures were more frequently recorded among spears (Table 1 and Figure 4C). Armatures hafted on spears delivered with crossbow on the other hand display a higher frequency of step fractures. However, these differences are not statistically significant whether we consider those recorded on armatures hafted on spears delivered with a crossbow (Fisher’s exact test, *p* value = 0.0617) or not (Fisher’s exact test, *p* value = 0.4545).

**Table 2:** Absolute and relative frequency of damage types observed on antler, bone, and ivory armatures

Damage type	Antler	Antler %	Bone	Bone %	Ivory	Total
Compression	13	31.71%	6	13.64%	–	19
Deformation	8	19.51%	1	2.27%	–	9
Split	1	2.44%	1	2.27	–	2
Tongue	–	–	5	11.36%	–	5
Tongue + compression	6	14.63%	4	9.09%	–	10
Double tongue	–	–	1	2.27%	–	1
Double tongue + compression	1	2.44%	–	–	–	1
Tongue + hinge	–	–	1	2.27%	–	1
Hinge	1	2.44%	2	4.55%	1	4
Hinge + compression	3	7.32%	5	11.36%	–	8
Step	3	7.32%	9	20.45%	1	13
Step + compression	4	9.76%	2	4.55%	–	6
Double step + compression	–	–	1	2.27%	–	1
Step + hinge	–	–	4	9.09%	–	4
Step + hinge + compression	–	–	2	4.55%	–	2
Sawtooth	1	2.44%	–	–	–	1
Total	41	–	44	–	2	87

See Data S1 for details.

**Table 3:** Absolute and relative frequency of damage types observed on large and small armatures

Damage type	Large	Large %	Small	Small %	Total
Compression	7	21.21%	12	23.08%	19
Deformation	5	15.15%	4	7.69%	9
Split	–	–	2	3.85%	2
Tongue	2	6.06%	3	5.77%	5
Tongue + compression	2	6.06%	8	15.38%	10
Double tongue	1	3.03%	–	–	1
Double tongue + compression	–	–	1	1.92%	1
Tongue + hinge	–	–	1	1.92%	1
Hinge	1	3.03%	2	3.85%	3
Hinge + compression	–	–	8	15.38%	8
Step	7	21.21%	5	9.62%	12
Step + compression	2	6.06%	4	7.69%	6
Double step + compression	–	–	1	1.92%	1
Step + hinge	4	12.12%	–	–	4
Step + hinge + compression	2	6.06%	–	–	2
Sawtooth	–	–	1	1.92%	1
Total	33	–	52	–	85

See Data S1 for details.

### Size and invasiveness of shooting damages

**Figure 5:**
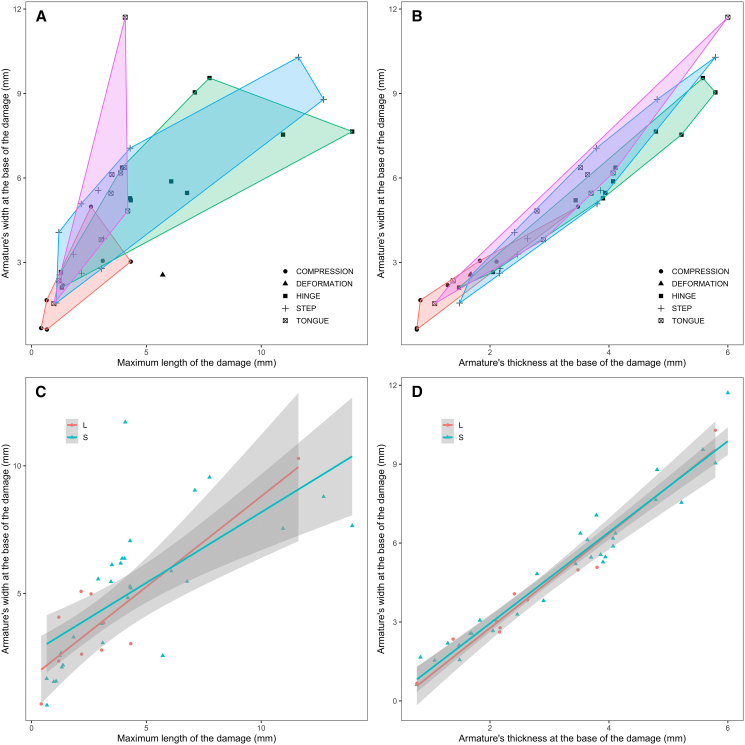
Relation between the armature’s width at the base of the distal damage (A and C) The maximum length of the damage and (B and D) the armature’s thickness at the base of the distal damage by (A and B) damage type and (C and D) armature’s size (L = large; S = small). (C and D) Linear trends and 95% confidence intervals by category. See Data S1 for details.

**Figure 6:**
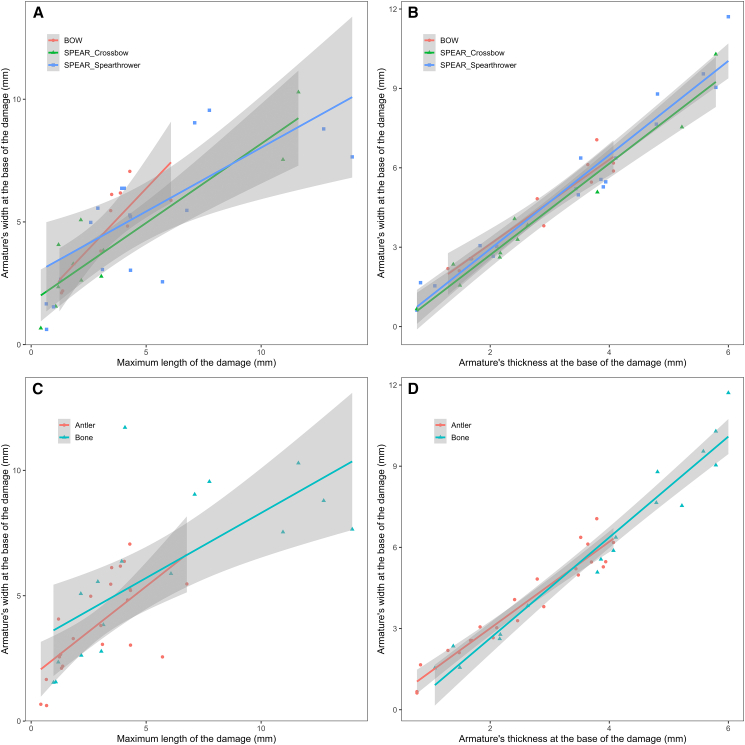
Relation between the armature’s width at the base of the distal damage (A and C) The maximum length of the damage and (B and D) the armature’s thickness at the base of the distal damage by (A and B) delivery method and (C and D) raw material. Linear trends and 95% confidence intervals by category. See Data S1 for details.

The size of the damages was investigated to establish whether differences in damage morphology also imply differences in their relative invasiveness or if they are determined by other factors. Significant differences were observed between the damage categories (PERMANOVA: *F*_(3,35)_ = 3.6687, *p* value = 0.007). Generally, tongue, hinge, and step damages show substantial overlap in their invasiveness with one another although tongue damages tend to display shorter lengths than hinge and step damages. Damage by compression is the least invasive of all (Figures 5A and 5B). The armature’s size (Figures 5C and 5D) and the delivery method (Figures 6A and 6B) do not appear to constitute determining factors to explain variations in damage invasiveness even though damages on armatures hafted on arrow tend to fall in the lower end of the range of variation recorded for spears. Interestingly, the range of variation recorded for spears delivered with spear-thrower and crossbow is relatively the same. Damages on antler points, however, tend to be less invasive than those recorded on bone armatures, which reflects the higher frequency of compression damage on antler points and suggests that raw material plays a determining role on how an armature responds to shocks (Figures 6C and 6D).

**Figure 7:**
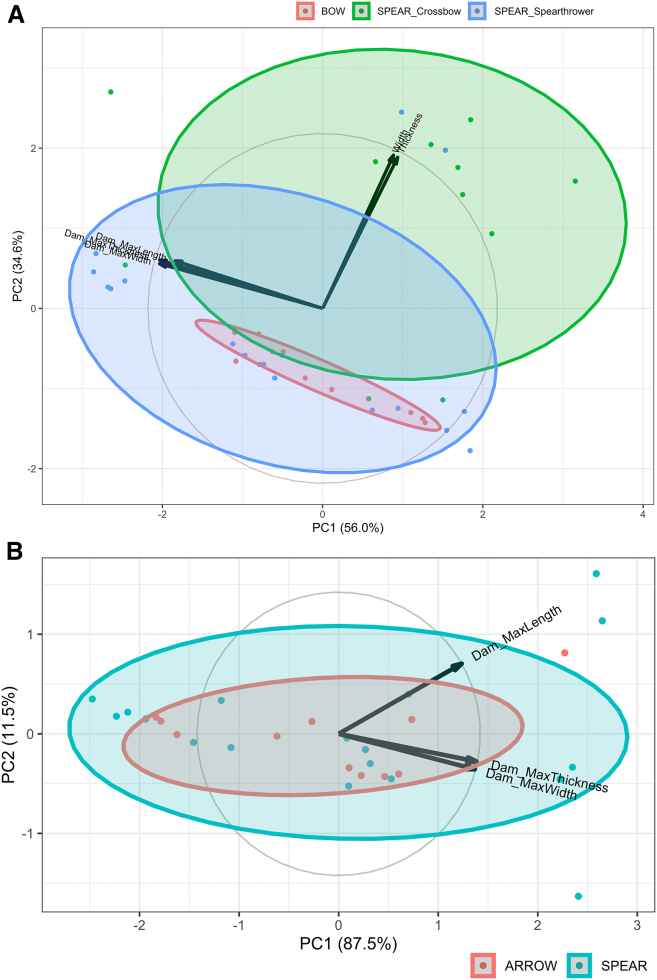
Principal component analysis of the metric data capturing the armature’s dimension (width and thickness) and the invasiveness of the distal damages it bears (Dam_MakLength, Dam_MaxWidth, and Dam_MaxThickness, the latter two being recorded at the base of the distal damage) (A) For all the projectile damaged in the course of the experiment. (B) Restricted to the small armatures hafted on either arrows or spears. Ellipses of confidence (95%) by projectile category. See Data S1 for details.

When the whole experimental sample is considered, the first two axes of the PCA comparing the damage invasiveness and the maximum width and thickness of the armatures, i.e., data that can be recorded on archaeological specimens, explain 90.6% of the variation (Figure 7A). The armature’s maximum width and thickness positively covary with PC1 and PC2 while the damage invasiveness variables covary negatively with PC1 and positively with PC2. Arrowheads fall within the range of morphometric variability recorded for spear points hafted on projectiles delivered with a spear-thrower. Most of the projectiles delivered with a crossbow yielded results that fall outside the variation recorded for arrows or spear delivered with a spear-thrower. When attempting to predict the projectile type from the size of an armature’s cross-section and the damage invasiveness, misclassification percentages recorded on the training sample ranged between 0% and 42% (μ = 26%, σ = 5%). After applying these models to classify the specimens included in the testing sample, the misclassification percentages rose drastically and ranged between 25% and 70% (μ = 40%, σ = 9%). This result entails that an individual is likely to misclassify the projectile type on average twice out of every five attempts when considering solely the armature’s size and damage invasiveness. Arrowheads are the most affected by misclassifications; 86% of the time (σ = 16%), they are wrongly interpreted as spear points. Likewise, spear points are conflated with arrowheads 19% of the time (σ = 17%).

When restricting ourselves to small armatures, the first two axes of the PCA comparing the damage invasiveness variables explain 98.95% of the variation (Figure 7B). The damage invasiveness variables all covary positively with PC1. The damage maximum width and thickness covary negatively while the damage maximum length covaries positively with PC2. Arrowheads fall within the range of morphometric variability recorded for spear points. When attempting to predict the projectile type from the damage invasiveness on small armatures, misclassification percentages recorded on the training sample ranged between 0% and 57% (μ = 33%, σ = 8%). After applying these models to classify the specimens included in the testing sample, the misclassification percentages rose drastically and ranged between 33% and 93% (μ = 55%, σ = 10%). This result entails that an individual is likely to misclassify the projectile type on average twice out of every five attempts when considering solely the armature’s size and damage invasiveness. Arrowheads are the most affected by misclassifications; 86% of the time (σ = 16%), they are wrongly interpreted as spear points. Likewise, spear points are conflated with arrowheads 35% of the time (σ = 22%). Thus, whether we consider split-based points or massive-based points of all sizes or restrict ourselves only to the small specimens, neither the damage type nor its invasiveness can reliably predict the projectile type on which they were hafted.

## Discussion

Identifying the weapons used by past humans is essential to fully appreciate how subsistence strategies were conceived and implemented, and to explore how they intersected with the environmental conditions, social organization, and group mobility. Documenting the role of osseous projectile points in prehistoric hunting strategies is becoming increasingly important as it has broad implications on our understanding of the successful dispersal of our species across the Old World. Increasing evidence suggests that *Homo sapiens* were established in various parts of Eurasia and Australia c. 55–40 ka following several early dispersals.^60^^,^^84^^,^^85^^,^^86^^,^^87^^,^^88^^,^^89^^,^^90^^,^^91^^,^^92^^,^^93^^,^^94^^,^^95^^,^^96^^,^^97^^,^^98^^,^^99^^,^^100^^,^^101^ The enduring colonization of new territories required adapting to new environments with varied—and often unique—flora, fauna, climate, and landscape. A critical aspect of this dynamic relates to the nature and efficiency of the hunting equipment. Indeed, projectile hunting weapons tipped with stone or osseous armatures certainly played a key role in successfully broadening the ecological niches exploited by prehistoric groups. The emergence of hunting strategies based on the use of more effective and safer technologies would have afforded for rapid population growth and extension of *H. sapiens*’ geographic range and social networks.^102^^,^^103^^,^^104^^,^^105^ Such extended diet breadths, regions, and networks are expected to vary in extent and character, reflecting climatic, demographic, and cultural factors.^106^ The variability of these niches—and their respective game resources and mobility strategies—argue against a linear and temporally constrained interpretation of all projectile technology.

In spite of the substantial efforts carried out over the last few decades, archaeologists are still forced to rely on indirect evidence, e.g., impact damage on armatures and their morphometry, to infer the types of weapons manufactured and used by Pleistocene human groups. The results of our shooting experiment contribute to filling a gap by making a comprehensive assessment on the possibility to discriminate spears and arrows among an assemblage of osseous projectile points. While other studies have tested this hypothesis for the projectiles from the Late Upper Palaeolithic (Magdalenian^28^), we assessed split-based points and massive-based points recorded at the beginning of the UP when the first *H. sapiens* permanently established themselves in Western Eurasia.

When the efficiency of Aurignacian armatures is considered, the kinetic energy of the projectile is not the only factor affecting its penetration depth. Our results lend further support to Knecht^107^^,^^108^ and Doyon and Knecht^63^ who, respectively, demonstrated that the size of the armature, particularly the protruding maximum width, and the nature of the impacted tissue play a significant role in the projectiles’ lethal capability, i.e., reach the internal organs and result in significant bleeding for the prey. The penetration depths reported here suggest that both spears and arrows would have been suitable weapons to inflict such a lethal wound to a prey. The sturdiness of both armature types was observed during the experiment when several specimens became lodged in bones during the experiment while they underwent limited damage, i.e., essentially compression. Furthermore, both armatures required a comparable number of shots before they sustained damages, which indicate that they are equally durable and can withstand multiple uses.

In terms of maintainability, our experimental results suggest that armatures hafted onto spears or arrows would have been as equally fit for prolonged use as long as the hafting itself could withstand repeated impacts without breaking. Irrespective of the weapon type, one-third of the recorded damages consists of compression, which entails no, or minimal, resharpening prior to repeated use. Although arrows appear to be more prone to tongue damage and spears to step damage, respectively, we could not demonstrate a significant relation between these armature types and the damage they bear.

**Figure 8:**
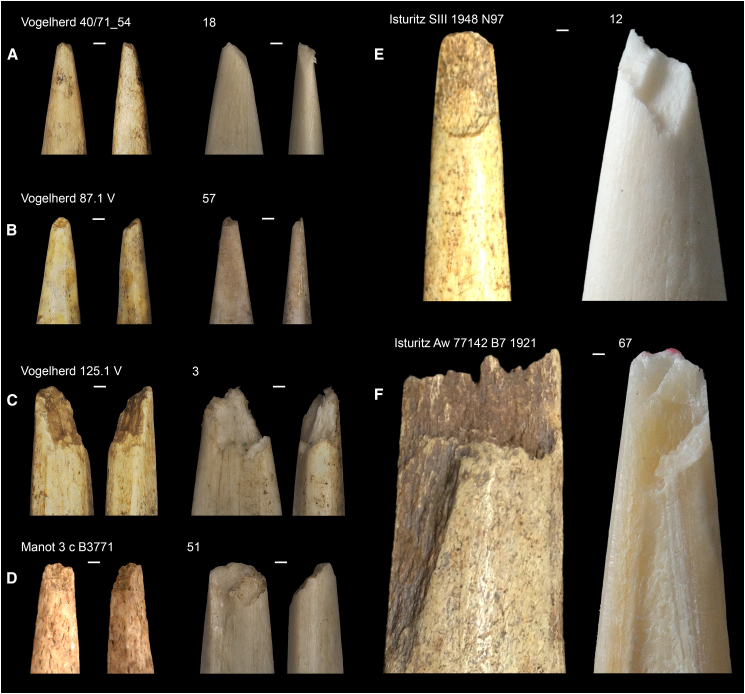
Archaeological examples from Aurignacian sites: Vogelherd (Germany), Isturitz (France), and Manot (Israel) compared with experimental specimens (A) Compression. (B) Tongue. (C) Compression + hinge-tongue. (D) Compression + hinge-tongue. (E) Step-tongue. (F) Step-tongue (with tongue and sawtooth breaks) are shown. Scales = 1 mm.

On the contrary, our results suggest that breakage patterns are more dependent on the raw material and size of the armature. Antler generally appears to be more resistant to impact forces than bone, which is coherent with the mechanical properties of this raw material^109^ and previous observations.^28^^,^^107^^,^^108^ In terms of damage invasiveness, aside from compression, tongue damage is generally shorter in length than hinge and step damage. Consequently, armatures affected by tongue damage would have had a greater potential to be maintained and reused than those presenting hinge and step damages. Interestingly, the damages recorded on our experimental specimens were observed in the archaeological record, specifically from several Aurignacian sites (Figure 8), which raises the question as to what factors influenced the decision process for prehistoric individuals to resharpen or discard their damaged armatures. Differences in the relative frequencies of the three invasive damage types in the archaeological record (e.g.,^65^^,^^68^^,^^80^^,^^81^^,^^110^^,^^111^) could indicate that once presenting a hinge or a step damage, prehistoric hunters may have favored to replace the broken armature rather than resharpening it. Given the significant association between the breakage patterns and the armature’s raw material and size, prehistoric groups may have also considered these factors when they strategized and manufactured their projectile points to minimize the impact of the damage and maximize the life expectancy of their weapons.

Our experimental results fundamentally question the relationship between the size of an osseous armature, the damage it bears or its invasiveness with the projectile type. The variability of damages observed on the experimental arrowheads is fully encompassed by the range recorded for damaged spear points delivered with a spear-thrower. This finding has important implications for our understanding of the osseous armatures of the Aurignacian and beyond.

It is generally assumed that the Aurignacian osseous armatures were hafted on spears delivered by spear-throwers. Alternative hypotheses in favor of arrows are found on the size of the armature^56^^,^^83^ and on the functional experiments that rely on too few specimens and offer no systematic comparison with other projectile types to reach definite conclusions (e.g.,^56^). Our results show that we cannot rule out points, which are delivered by bows, when we interpret the osseous projectile points nor assume that all of them were delivered using spear-throwers. Although the armature’s maximum thickness would have constrained the diameter of the projectile shaft or foreshaft,^65^^,^^68^^,^^112^ the small size of a specimen is not a sufficient criterion to make a reliable inference on the projectile type. As such, the choice of spears versus arrows would likely have been conditioned by, among other, the availability and quality of the wood resources in the environments occupied by Aurignacian hunters.

Our results also question the traditionally preferred scenario to account for the linear evolution of Pleistocene hunting weapons. In the absence of direct evidence such as spear-thrower hooks or perishable components of bow-and-arrow technology, it is difficult to infer with certainty the projectile type from the discarded osseous armature alone.

To this end, many parameters influence the composition of the hunting toolkit. Environmental factors include the availability of plant species, the targeted prey’s size, habitat, availability, and mobility^113^^,^^114^^,^^115^^,^^116^^,^^117^^,^^118^^,^^119^^,^^120^^,^^121^^,^^122^ while socioeconomic and cultural factors comprise the patterns of group and individual mobility, the technological system, the division of labor and craftsmanship as well as the shared belief system that make sense of the relations between these parameters.^14^^,^^18^^,^^59^^,^^103^^,^^104^^,^^123^ Several ethnographic examples demonstrate that hunting toolkits may indeed combine a variety of weapon types.^124^^,^^125^^,^^126^^,^^127^^,^^128^^,^^129^ Similar adaptations cannot be excluded for the second half of the Upper Pleistocene, and seem in fact supported by the emergence of bow-and-arrow technology in the African MSA,^19^^,^^47^^,^^48^^,^^49^^,^^51^ and the introduction of new raw material such as antler for the manufacture of projectile points from the onset of the Upper Palaeolithic elsewhere.^65^^,^^80^^,^^81^

It appears reductive to assume that the penecontemporaneous emergence of osseous projectile points in various regions of the Old World implies that populations living in various environments converged in opting for the same weapon type. The diversity in armature’s shape, especially when the proximal hafting portion is considered, suggests that a diversity of hafting mechanisms began to be manufactured circa 45–40 ka. These innovations may also indicate that launching mechanisms were equally diversified. The coexistence of lithic and osseous armatures throughout the Upper Palaeolithic likely testifies to a trend toward an increased reliance on a multiplicity of hunting technologies. Indeed, osseous armatures present several advantages over lithic ones. Generally, osseous raw materials are more easily accessible as they can be acquired during prey procurement activities, although some raw material procurement such as shed antler collection may have relied on particular strategies akin to lithic procurement.^111^ Furthermore, their properties allow the production of objects on which a shape can be imposed with a high degree of precision with adapted techniques such as grinding, scraping, and gouging. Finally, the composition and structure of osseous materials^109^ afford the manufacture of lightweight standardized implements that are durable, maintainable (sensu^113^^,^^114^), and can be assembled into multi-component complex technologies. By increasing the number of options an individual could carry and choose from, Palaeolithic group members were potentially afforded with the possibility to heighten their mobility to carry out their subsistence activities while increasing their success rate in prey procurement.

Our study in part demonstrates the complex nature of reconstructing projectile technology, which is often created with perishable materials. While it is impossible to account for all variables that affect the physical properties of the armatures and the resulting damages, a series of experimental programs that is designed to address multi-faceted nature of projectiles in the future can hopefully shed more light onto one of the important pillars of hunter-gatherer’s economy.

### Limitations of the study

This experimental study acknowledges several methodological constraints that, while important to note, do not compromise the validity of our main conclusions. First, the calibrated crossbow employed to standardize shooting parameters in the first experimental session (September 2022), while differing from prehistoric weapon launch dynamics with spear-throwers and bows, served its intended purpose of providing controlled, replicable conditions. Subsequent manual shooting experiments confirmed that experienced individuals deliver significantly higher kinetic energy values, demonstrating that our crossbow-delivered projectiles represent the lower end of the efficiency spectrum and thus provide conservative estimates.^130^^,^^131^ This methodological choice strengthens rather than undermines our conclusions regarding projectile effectiveness.

Second, ballistic gel targets employed in the second session (July 2023), despite not fully replicating the biomechanical properties of animal muscle and soft tissue, provided standardized conditions that facilitated systematic comparative analysis. The bimodal distribution of penetration depths observed when projectiles impacted bones encased in ballistic gel contrasts with the continuous distribution recorded for shots on animal carcasses,^130^^,^^131^ suggesting that the patterns observed in actual hunting scenarios would be even more favorable. Our results from animal carcasses, though limited in number, support the patterns identified and validate the broader conclusions drawn from the ballistic gel experiments.

Third, while the number of arrow shots performed on animal carcasses was limited, the available data are consistent with the patterns observed in the larger experimental sample and support our interpretations regarding the efficiency of both arrows and spears. The consistency of damage patterns across different target types reinforces confidence in our findings.

Importantly, our core finding that breakage patterns depend more on the raw material and size of the armature than on the specific launching mechanism remains robust across all experimental conditions. The substantial overlap in damage types and invasiveness between arrows and spears, documented across 191 shots with 60 projectiles, provides a solid empirical foundation for our conclusions. Future experimental programs incorporating additional controlled variables and larger sample sizes on animal targets will further refine these interpretations and expand our understanding of Aurignacian projectile technology.

## Resource availability

### Lead contact

Further information and requests for resources should be directed to and will be fulfilled by the lead contact, Keiko Kitagawa (keiko.kitagawa@uni-tuebingen.de).

### Materials availability

**Figure 9:**
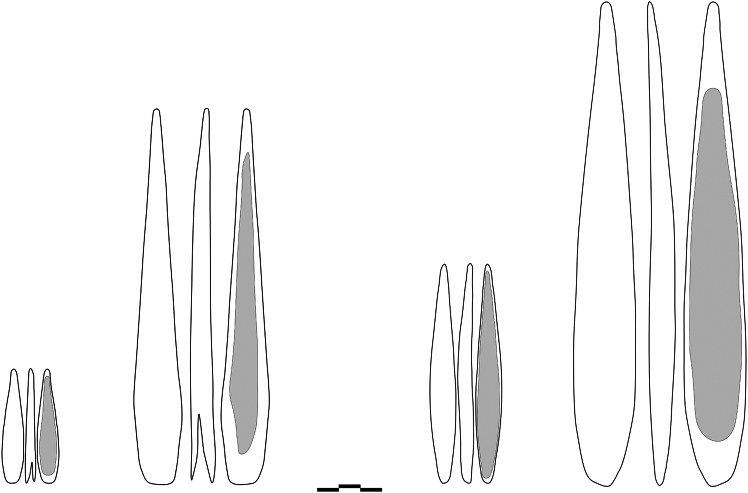
Outline of points and dimensions (maximum length × maximum width × maximum thickness) From left to right: small split-based point from Arbreda (50 mm × 9.9 mm × 3.7 mm), large split-based point from Gorge d'Enfer (168.9 mm × 22.2 mm × 10.7 mm), small massive-based point from Mallaetes (125.5 mm × 15.3 mm × 8.4 mm) and large massive-based point from Mladeč (179 mm × 27.8 mm × 12.1 mm). Note: gray area represents the spongiosa/cancellous areas. Scale = 3 cm.

All experimental osseous tools that were analyzed in this study are curated in the Senckenberg Center for Human Evolution and Palaeoenvironment/Early Prehistory and Quaternary Ecology Working Group, Department of Geosciences, University of Tübingen under the accession code TUE_AURPP. The archaeological specimens illustrated in Figure 9 are curated in the following institutions and with the following accession code: University of Tübingen (Figure 9A: accession # 40/71_54; Figure 9B: accession # 87.1V; Figure 9C: accession # 125.1V); Israel Antiquities Authorities (Figure 9D: accession # 3 c B3771); Musée d’Arqueologie Nationale de Saint Germain-en-Laye (Figure 9E: accession # SIII 1948 N97; Figure 9F: accession # Aw 77142 B7 1921).

### Data and code availability

The experimental projectile points used in this study can be physically accessed at the University of Tübingen upon request. In addition, the essential raw quantitative data are found in the supplemental table (SOM 1), and if needed, additional quantitative data can be shared upon request. Lastly, the codes employed existing codes. Therefore, codes are not deposited but it can be shared upon request by contacting Luc Doyon (luc.doyon@u-bordeaux.fr).

## Acknowledgments

For their support and assistance on aspects of this research, we are indebted to our colleagues at the Senckenberg Centre for Human Evolution and Palaeoenvironment and 10.13039/501100002345University of Tübingen, especially N.J. Conard, B. Starkovich and S. Wolf, and to the “Lignées humaines, cultures, environnements du Paléolithique ancien au début du Paléolithique récent en Eurasie (EURAPAL)” team, CNRS UMR5199 PACEA. We also thank Pierre Lansac for sharing his knowledge on prehistoric projectile weaponry and recommending insightful ethnographic literature. This research was funded by the 10.13039/100005966Leakey Foundation (research grant S202210320 to K.K.); the Département des Sciences archéologiques, 10.13039/501100006251University of Bordeaux (research grant AAP n˚4-SaFlèch to L.D.); and benefited from the scientific framework of the University of Bordeaux’s IdEx “Investments for the Future” program/GPR “Human Past” (L.D.) and the European Research Council Starting Grant for the project Pleistocene Expedient Osseous Technology (ExOsTech, no. 101161065) (L.D.). J.-M.T.’s research is supported by a project of the Meitner Program of the 10.13039/501100002428Austrian Science Fund (FWF) (project: Osseous Hunting Weapons of Early Modern Humans in Eurasia #M3112), the program Ramón y Cajal (grant RYC2021-033759-I funded by MICIU/AEI/10.13039/501100011033 and by European Union NextGenerationEU/PRTR), and the SERP of the University of Barcelona (SGR: 2021SGR_00337). We thank the Editor and reviewers for their comments that contributed to improving this manuscript. Open access funding was provided by the 10.13039/501100003065DEAL Agreement.

## Author contributions

K.K., J.-M.T., and L.D. contributed to the conception; K.K., J.-M.T., L.D., R.Y., and R.W. designed the work; K.K., J.-M.T., L.D., R.Y., R.W., H.H., R.A., and N.M. data acquisition and analysis; K.K., J.-M.T., and L.D. funding acquisition; K.K. and L.D. project administration; K.K., J.-M.T., L.D., R.Y., and R.W. interpreted the data; L.D., H.H., R.A., J.-M.T., and K.K. visualization; K.K., J.-M.T., L.D., R.Y., R.W., H.H., R.A., and N.M drafted and revised the manuscript; K.K., J.-M.T., L.D., R.Y., R.W., H.H., R.A., and N.M approved the submitted version.

## Declaration of interests

The authors declare that they have no competing interests.

## STAR★Methods

### Key resources table

**Table:** 

REAGENT or RESOURCE	SOURCE	IDENTIFIER
**Deposited data**		

Experimental projectile points made of bone, antler, and ivory	This study	Data S1
Archaeological projectile points made of antler found at Vogelherd	University of Tübingen, Germany	Accession # 40/71_54, 87.1V, and 125.1V
Archaeological projectile point made of antler found at Manot 3	Israel Antiquities Authorities, Israel	Accession # 3 c B3771
Archaeological projectile points made of antler found at Isturitz	Musée d'Archéologie Nationale de Saint Germain-en-Laye, France	Accession # SIII 1948 N97, and Aw 77142 B7 1921

**Software and algorithms**		

RStudio	RStudio Team https://posit.co/	Version 2024.12.1+563
R (scripts available upon request, no unique R codes were established)	R Core Team https://cran.r-project.org/	Version 4.4.1

#### Experimental model and study participant details

The antlers, bones, and carcasses used in the present study were obtained through local hunters, butchers, and farmers (Schelklingen, Germany) in compliance with local regulation. No animals were killed or injured specifically for the purpose of the study. All experimental samples were curated in Tübingen, Germany, in sterile plastic bags and given unique specimen identifiers.

### Method details

#### The projectile manufacture

In total, 60 out of 102 armatures were used for the present project, including split-based points in reindeer antler, massive-based points in horse metapodial bones, and massive-based points in ivory. In addition, antler and bone points were each divided into two groups of small and large armatures. The manufacture of the armatures involved the use of electric tools for the production of blanks and preforms. The final shaping of the replicas was done with retouched lithic tools made from flint. The size and morphology of the small armatures were based on a split-based point from Cova de l’Arbreda and a massive-based point from Les Mallaetes (Spain) while the large ones are a replica of split-based point from Gorge d’Enfer (France) and massive-based point of Mladeč (Czech Republic) respectively^68^^,^^80^ (Figure 9). The small split-based points measured on average 55 mm in length, 12 mm in width, and 6 mm in thickness. Their split length was 11 mm on average and their weight averaged 3 g. The large split-based points measured on average 172 mm in length, 22 mm in width, and 11 mm in thickness. Their split length was 25 mm on average and their weight averaged 24 g. The small bone massive-based points measured on average 103 mm in length, 12 mm in width, and 6 mm in thickness. Their weight averaged 6 g. The large bone massive-based points measured on average 174 mm in length, 24 mm in width, and 9 mm in thickness. Their weight averaged at 36 g.

The two ivory massive-based points were shaped based on specimens found at Hohle Fels (Germany). They measured on average 147 mm in length, 9 mm in width, 9 mm in thickness, and weighted on average 14 g.

A total of 60 armatures, i.e., 22 small and 7 large split-based points, 19 small and 10 large bone massive-based points and 2 ivory massive-based points, were hafted on pine dowels measuring 9 mm for the arrow shafts (n = 19) and 13.5 mm for the spear foreshafts (n = 41). While we hafted both small and large split-based points and massive-based points on spear foreshafts, only small split-based points and massive-based points were hafted on arrow shafts. A U-shaped slot was cut at the distal end of each dowel following descriptions by Knecht^107^^,^^132^ with a bandsaw and gouged with wood scissors before being thinned with sandpaper to limit the bulging of the hafting area and ensuring the junction between the armature and the projectile shaft was as streamlined as possible. The armatures were inserted in the slots and their position was secured with a mixture of hide glue, charcoal and ochre; wedges were inserted in the split of the antler points to spread their proximal tongues apart. Synthetic sinew was used to wrap the hafting area tightly and beeswax was applied to smooth the hafting. Arrows and spear shafts were fletched with turkey feathers and synthetic sinew. The complete arrows and spears weighted on average 28 g and 217 g respectively.

#### The shooting experiment

The shooting experiment was performed in Schelklingen, Germany, over two sessions in September 2022 and July 2023. In September 2022, two animal targets were used, i.e., a one-year-old subadult female roe deer (*Capreolus capreolus*) and a five-year-old female sheep (*Ovis aries*). In this session, the projectiles were mainly delivered with a hand-made crossbow calibrated at a draw weight of ∼27 kg, the maximum draw weight possible with the crafted crossbow and, in some instances, manually with a spearthrower and a traditional bow by an experienced individual (RW). Several shots were also performed by an inexperienced spearthrower during this session (JMT). In July 2023, two blocks of ballistic gel covering bovid long bones and scapulae were used as targets. Upon realising that the speed of the shots was slower on average for the crossbow than those delivered manually with a spearthrower and a traditional bow, all shots in this session were performed by Joachim Martz, an experienced participant in European prehistoric shooting contests using a spearthrower and bow. Projectiles were shot at a distance ranging between 10 and 13 m from the targets, which exploited the maximum distance available in the field where the experiment was conducted. Following each hit, we recorded the anatomical location of the hit and the bone(s) that came into contact with the projectile by manual examination, the depth of penetration of the projectile and any damage to the armature and/or haft (see^133^ for an analysis of the projectile impact marks on bone and the anatomical distribution of the hits). When shot on ballistic gel blocks, the distinction was only made between gel and bone in lieu of the anatomical location of the hit. The description of damage types followed the terminology by Pétillon.^28^ Each projectile was repeatedly shot until it was no longer usable, either because of tip and/or hafting damage or when the point got stuck in the bone. This meant that points which ended up hitting the ground or missed the target were repeatedly used thereafter as long as shots did not lead to sustained damage (see also SOM 1).

#### Handling outliers

Several outliers were removed from the ballistic analysis; they nonetheless appear in the supplementary data (Data S1). The penetration data for point #71, shot “F”, is not considered because the projectile penetrated the ballistic gel at a depth >1 m, which seems unlikely in a real-life setting. It was also decided not to consider the data recorded for the ivory massive-based points because of the small sample size for this raw material. Finally, shots delivered by the inexperienced spearthrower were also discarded because the projectiles rarely hit the target, and when they did, they bounced back upon contact with the skin owing to insufficient kinetic energy to penetrate it.

#### The ballistic analysis

The efficiency of the projectile was explored through the depth of penetration of the projectile relative to its kinetic energy to establish whether it would have induced a lethal wound to the prey. Although penetration, in and of itself, is not the only factor that could induce a lethal wound, it is an adequate proxy to establish whether a projectile lacking lacerating properties could reach the vital organs of the prey and cause significant bleeding.^107^^,^^132^ The number of shots and the frequency and type of damage per shot was also quantified to appreciate the durability of the armature.

To demonstrate whether it is possible to infer the nature of the projectile from the type of damage on its armature, several quantitative variables were recorded, i.e., the maximum length of the damage, the width and thickness of the armature at the base of the damage as well as the maximum width and thickness of the armature. These variables were selected because they can easily be reproduced by distinct individuals and correspond to data that can be recorded on archaeological specimens.

#### Imaging methods

All armatures were photographed prior and after their use with a Nikon D7200 with Sigma 17 - 70 F2.8-4 DC Makro OS HSM. Tip damage was recorded with a Hirox Digital Microscope HRX-01 with lens HR-2500 and recorded at FOV of 15.2 mm (20x magnification). During the shooting experiment, the shots were recorded with a high-speed camera Photron Fastcam Mini AX 200 Tamron SP 24–79 mm to calculate the projectile’s speed and kinetic energy (J).

### Quantification and statistical analysis

Statistical analyses were performed after discarding the outliers. They, along with their respective graphic representations, were all performed in RStudio v2024.12.1+563 running R-CRAN v4.4.1.^134^ For statistical tests, exact values of *n*, i.e., number of projectile points or damages, were used and dispersion measures were presented. Significance was established based on α ≤ 0.05 for the Student’s *t* tests, ANOVAs and Tukey’s HSD post-hoc tests, Pearsons’s *t* tests, Chi-squared tests, and Fisher’s exact tests to compare the efficiency of the projectile based on the delivery method, armature’s size, point type and impacted tissue as well as the shooting damages based on the raw material of the armature, its size, and the projectile type. PCA analysis were computer after normalising all variables and centring their values around zero. Ellipses were computed at a 95% confidence interval.

To test whether it was possible to predict the projectile type from the PCA’s eigenvalues, our experimental sample was subdivided into two groups, i.e., a training group and a testing group. In the training group, we randomly selected half of the arrowheads and of the spear points. The remaining specimens were assigned to the testing group. A model was first calculated on the training group, then applied to the testing group, and the percentage of misclassification was calculated from the resulting confusion matrix. This process was repeated over 10,001 iterations to quantify the performance of the predictive models. The PCA and discriminant analysis were performed first on the whole experimental sample and then restricted to small armatures. This second batch of multivariate analysis was justified because the size of the armature determines the diameter of the projectile shaft. Consequently, hafting large points on arrows was not possible. It was therefore relevant to establish whether some distinctions could be made as to the projectile type for a subset of our sample size. However, given their small size, the variables used for the PCA were restricted to those related to damage invasiveness, or the extent of damage on a given armature.

## Supplemental information

Data S1. Supplementary table with the details of each experimental shot including the raw material of the point, delivery system of projectile point, speed, target, penetration depth, dimension of point, breakage pattern, and the dimension of the breakage
